# Structural analysis of haemoglobin binding by HpuA from the *Neisseriaceae* family

**DOI:** 10.1038/ncomms10172

**Published:** 2015-12-16

**Authors:** Chi T. Wong, Yingqi Xu, Akshari Gupta, James A. Garnett, Steve J. Matthews, Stephen A. Hare

**Affiliations:** 1 Department of Life Sciences, Imperial College London, South Kensington Campus, Exhibition Road, London SW7 2AZ, UK

## Abstract

The *Neisseriaceae* family of bacteria causes a range of diseases including meningitis, septicaemia, gonorrhoea and endocarditis, and extracts haem from haemoglobin as an important iron source within the iron-limited environment of its human host. Herein we report crystal structures of apo- and haemoglobin-bound HpuA, an essential component of this haem import system. The interface involves long loops on the bacterial receptor that present hydrophobic side chains for packing against the surface of haemoglobin. Interestingly, our structural and biochemical analyses of *Kingella denitrificans* and *Neisseria gonorrhoeae* HpuA mutants, although validating the interactions observed in the crystal structure, show how *Neisseriaceae* have the fascinating ability to diversify functional sequences and yet retain the haemoglobin binding function. Our results present the first description of HpuA's role in direct binding of haemoglobin.

## Abstract (web summary)

The *Neisseriaceae* bacteria family extract heame from the haemoglobin of its host, the HpuA protein is part of this system. Here, the authors report crystal structures of apo- and haemoglobin-bound HpuA and analyse mutants to examine the interaction between HpuA and haemoglobin.

## Graphical abstract

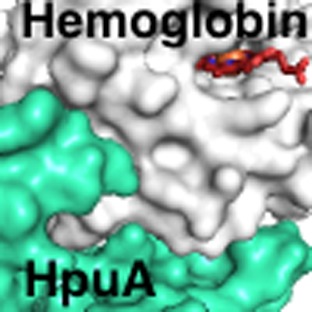

Bacteria have evolved elaborate iron uptake systems to overcome the scarcity of bioavailable iron, a problem particularly acute for pathogenic species when invading hosts where the free iron concentration is further limited as part of the innate immune system^1^. Most of the iron in mammals is stored intracellularly in haemoglobin (Hb) and ferritin complexes, and any free iron in serum and other bodily secretions is quickly sequestered by transferrin and lactoferrin, respectively. Almost two thirds of all iron in the human body is stored in tetrameric Hb inside red blood cells; on haemolysis, released Hb dissociates into dimers and is bound by haptoglobin (Hp) with very high affinity^2^. Hp serves two functions: to protect the body from the oxidative properties of Hb and to facilitate its removal by macrophages^3^,^4^,^5^. The *Neisseriaceae* family of Gram-negative bacteria express a bipartite receptor, HpuAB, to efficiently use haem extracted from Hb:Hp complexes as an iron source^6^,^7^.

The *Neisseriaceae* family is human host specific and includes the pathogenic *Neisseria* strains *Neisseria meningitidis*, the primary cause of bacterial meningitis and septicaemia, and *N. gonorrhoeae*, the causative agent of gonorrhoea. Systemic meningococcal infections continue to be a major health concern worldwide, causing death in up to 10% of cases and at times leaving survivors with permanent disability^8^. Gonococcal infections, with potential to cause pelvic inflammatory disease and infertility, are on the rise worldwide and resistance is emerging to all classes of antibiotic. Without the development of new therapeutics it is likely to be that *N. gonorrhoeae* will soon become an untreatable superbug^9^,^10^. Other pathogenic HpuAB-containing genera of the *Neisseriaceae* are *Kingella* and *Eikenella*, which, similar to *N. meningitidis*, are commonly carried asymptomatically in the oropharynx^11^,^12^. *Kingella* species, the most common of which is the important emerging paediatric pathogen *Kingella kingae*, cause osteoarticular infections such as septic arthritis or spondylodiscitis^13^. *Eikenella corrodens* also causes osteomyelitis and acute infection is most commonly observed in human bite or clenched fist injuries^14^. Both *E. corrodens* and *Kingella* species also form part of the HACEK (*Haemophilus*, *Aggregatibacter*, *Cardiobacterium*, *Eikenella*, *Kingella*) group of Gram-negative bacteria causing infective endocarditis^15^.

Of the two Hb receptors identified in *Neisseria*, HmbR and HpuAB, only the HpuAB system is capable of extracting haem from Hb in complex with Hp; it is also the only functional Hb receptor in published *N. gonorrhoeae* sequences, which contain a premature stop codon in HmbR^6^,^16^,^17^,^18^. Although found at variable frequencies in meningococci, Tauseef *et al*.^19^ discovered that 99% of disease-associated meningococcal strains encode one or both Hb receptor systems and, for meningococcal clonal complexes causing the highest rates of disease, 90% contain both HpuAB and HmbR. HpuAB is phase variable and, interestingly, in *N. gonorrhoeae* isolated from female patients HpuAB expression was more likely to be induced during early menses^20^,^21^. For meningococcal expression, it was found that one or both receptors were turned on in 91% of meningococcal disease isolates and 71% of carriage isolates, suggesting a link between Hb utilization and disease^19^,^22^. The importance of Hb exploitation was also observed in an infant rat model of meningococcal infection, where an HmbR knockout mutant of *N. meningitidis* was attenuated^16^.

The HpuB protein resembles TonB-dependent receptors and has been shown to be sufficient for binding of Hb at a low affinity by *Neisseria* but not for import of haem^23^. HpuA is expressed outside the cell, anchored to the outer membrane by its acylated amino terminus^7^. Although a direct interaction between HpuA on meningococci and Hb was not detected using flow cytometry, the lipoprotein is required for the high-affinity interaction between HpuB and Hb, for meningococcal survival on Hb as a sole iron source and for efficient dissociation of Hb^20^,^23^. Growth is permitted using Hb from a range of different mammals as an iron source^24^. An investigation into the vaccine potential of HpuAB revealed that sequences of both proteins from a range of *Neisseria* species are under strong immune selection^25^.

Some homology exists between HpuAB and the other bipartite iron receptors of *Neisseria*, the transferrin receptor TbpAB and lactoferrin receptor LbpAB. The best studied of these systems, the transferrin receptor, includes TbpB, a bilobed extracellular lipoprotein about twice the size of HpuA and sharing ∼17% identity. However, in contrast to HpuA, which is insufficient for binding Hb but essential for survival on Hb as a sole iron source, TbpB binds iron-loaded transferrin with high affinity and yet in some cases *in vitro* iron import from transferrin is possible by meningococci expressing the TonB-dependent receptor TbpA in the absence of TbpB, albeit at a reduced efficiency^26^.

Although the structure–function relationship of transferrin receptors has been investigated in detail^27^,^28^,^29^, no similar description is available for the Hb receptors and many basic questions remain as to the mechanism of haem uptake. In this study we report the structure of HpuA and reveal for the first time a direct interaction with Hb. Furthermore, we present the high-resolution structure of the HpuA complex with Hb (the first report of the structure of a Gram-negative receptor–Hb complex) and demonstrate that this complex is conserved in HpuA proteins from diverse *Neisseriaceae* family members. Further advancing our knowledge of the structure–function relationship of these receptors will allow for rational approaches in the design of future vaccines based on outer membrane and surface-exposed proteins.

## Results

### Structural description of HpuA

**Figure 1:**
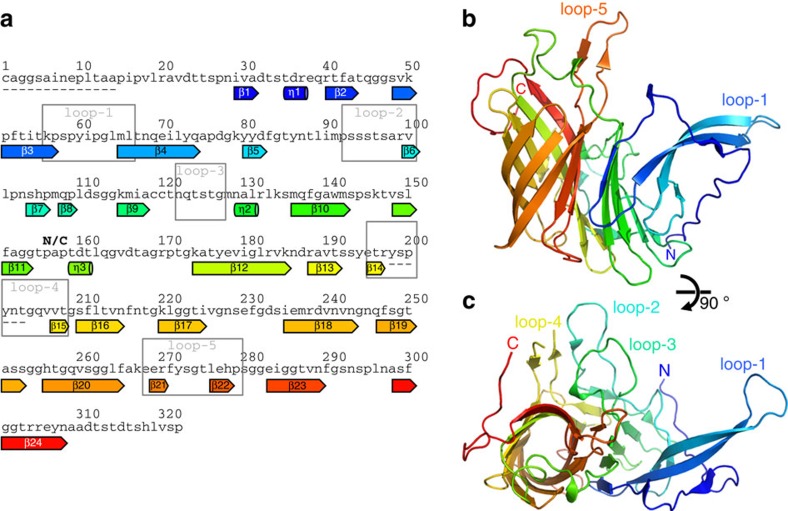
Structural features of KdHpuA. (**a**) The amino acid sequence of KdHpuA with secondary structure elements (identified by Stride^68^) labelled beneath and coloured from blue at the N terminus to red at the C terminus, and boxes indicating the loop regions mentioned in the text. Portions of the sequence not visible in the electron density are indicated by dashes beneath the sequence. (**b**) Cartoon representation of the structure showing secondary structure elements (coloured as in **a**) and labelling the prominent loops, as well as the N and C termini. (**c**) A 90° rotation of **b** about the *x*-axis, allowing visualization of loops 2, 3 and 4.

To investigate the structure and function of HpuA we crystallized the carboxy-terminal portion of the protein from *N. gonorrhoeae* (NgHpuA-C) and a full-length homologue from *K. denitrificans* (KdHpuA). The two homologues, KdHpuA and NgHpuA, share 30% sequence identity and 48% similarity. We solved the KdHpuA structure by single wavelength anomalous dispersion (SAD) following soaking crystals in cobalt ions (Table 1). Residues from 15 to 197 and 204 to 322 are visible in the electron density (Supplementary Fig. 1a,b and Fig. 1). The structure reveals a single domain composed of a small compact C-terminal β-barrel (residues 157–322) and a more open N-terminal β-sandwich (residues 1–156) (Fig. 1). Prominent features of the structure include two long extensions from the core of the protein, one formed from two longer strands of the N-terminal β-sandwich (β3 and β4) (loop-1) and the second from a loop at one end of the C-terminal β-barrel, between strands β20 and β23 (loop-5) (Fig. 1). Both loops present hydrophobic side chains at their distal end including those of Tyr-60, Phe-271 and Tyr-272. A third long loop dividing the second strand of the β-barrel (into β13 and β16) is partially disordered in the structure but also contains the hydrophobic residue Tyr-201 (loop-4). Two shorter loops on the same side of the protein comprise residues 92–100 (loop-2) and 122–127 (loop-3) (Fig. 1).

**Figure 2:**
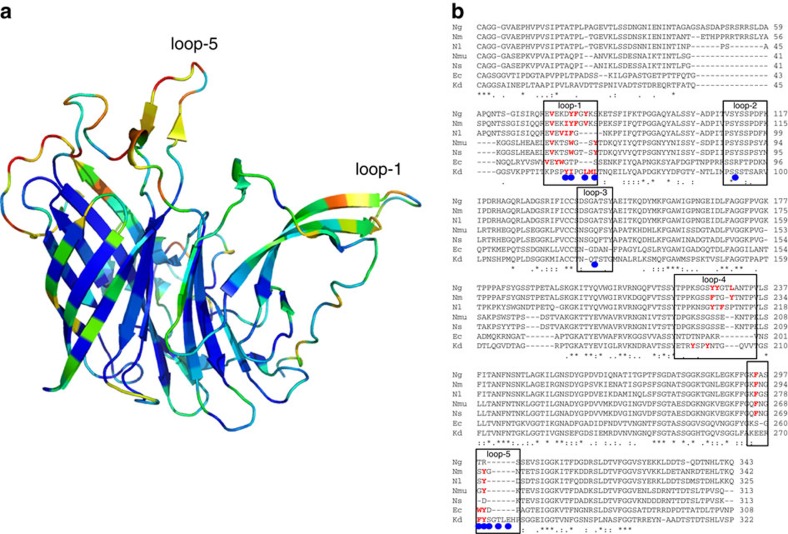
Conservation among HpuA homologues. (**a**) Cartoon representation of KdHpuA (oriented as in Fig. 1b) with residues coloured according to sequence conservation: blue for the most conserved, through to red for the least conserved. Conservation scores generated by Consurf^69^. (**b**) Sequence alignment of HpuA homologues from *N. gonorrhoeae* (Ng), *N. meningitidis* (Nm), *Neisseria lactamica* (Nl), *Neisseria mucosa* (Nmu), *Neisseria sicca* (Ns), *E. corrodens* (Ec) and *K. denitrificans* (Kd). Boxes enclose the loop regions mentioned in the text, blue circles beneath the alignment indicate KdHpuA residues observed to interact with Hb and hydrophobic side chains on loop-1, loop-4 and loop-5 are coloured red.

The NgHpuA-C structure was solved by molecular replacement using KdHpuA residues 168–322 as a search model. The NgHpuA-C structure is almost identical to the C-terminal β-barrel of KdHpuA (root mean squared deviation of 1.28 Å for 120 Cα atoms) with the only variation being the loop between β23 and β24 where NgHpuA-C includes an extra 3_10_ helix (Supplementary Fig. 2). A full-length model of *N. gonorrhoeae* HpuA was constructed by combining a Phyre2 (ref. 30) homology model (based on KdHpuA) with the NgHpuA-C crystal structure. A comparison of KdHpuA and NgHpuA sequences reveals significant differences between the loop regions (Fig. 2). NgHpuA has a three-residue insertion in loop-4 and a six-residue deletion at the site of loop-5. Inspection of sequences of HpuA homologues from other *Neisseria* and members of the wider *Neisseriaceae* family reveal that the highest degree of conservation is present in the central core of the protein, while the long extended loops are the least conserved (Fig. 2). This is in agreement with recent sequence analysis of HpuA from various *Neisseria* strains and suggests these regions are exposed to antibody selection^25^. Remarkably, although the long loops (loop-1, loop-5 and, to a lesser extent, loop-4) are poorly conserved and solvent exposed, they possess prominent hydrophobic side chains in all homologues (highlighted red in Fig. 2b).

Searching the protein data bank for related structures using the DALI server^31^ reveals the closest structural homologue is the C-terminal lobe of the TbpB component of transferrin receptor also found in *Neisseria* (Supplementary Fig. 3). Other extracellular lipoproteins of *Neisseriaceae* (Factor H-binding protein, NhbA and the N-terminal lobe of TbpB) have also been observed to adopt a similar overall fold with C-terminal small β-barrels and less compact N-terminal sheet regions (Supplementary Fig. 3)^27^,^28^,^32^,^33^. Notably, in TbpB, although the C-lobe structure is closer to that of HpuA, it is the N-lobe that is responsible for the direct binding to iron-loaded human transferrin.

### HpuA interacts directly with human Hb

**Figure 3:**
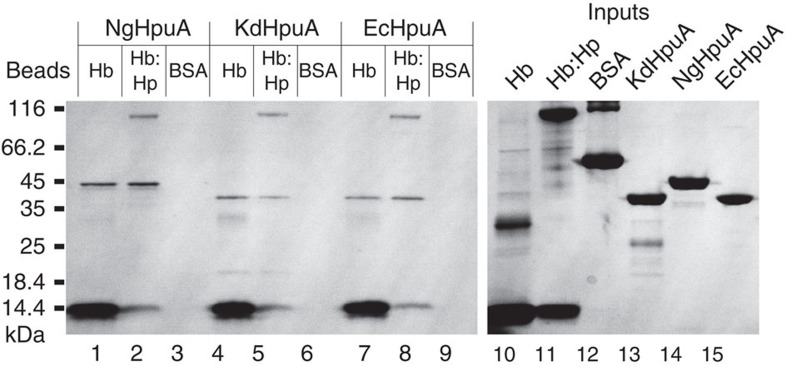
Haemoglobin can pull down various HpuA homologues. Coomassie-stained gel showing Hb, Hb:Hp and albumin control (BSA) beads pulling down KdHpuA (lanes 1–3), NgHpuA (lanes 4–6) and EcHpuA (lanes 7–9) proteins. Binding reactions were carried out in 500 ml solutions containing 3 μM HpuA. Input samples of all proteins used are shown in lanes 10–15.

Pulling down HpuA (from a 3-μM solution) with Hb cross-linked agarose resin demonstrates that there is a weak direct interaction between the two proteins (Fig. 3). This is in contrast to previous results using HpuA on the surface of *N. meningitidis* that failed to detect any direct interaction, although it should be noted that the detection limit in this assay was quoted as a dissociation constant of 5 μM (ref. 23). Immobilized Hb or Hb:Hp complex pulled down wild-type KdHpuA and NgHpuA, as well as a divergent homologue from *E. corrodens* (EcHpuA) (Fig. 3). Similar pull down was not observed using BSA control beads.

**Figure 4:**
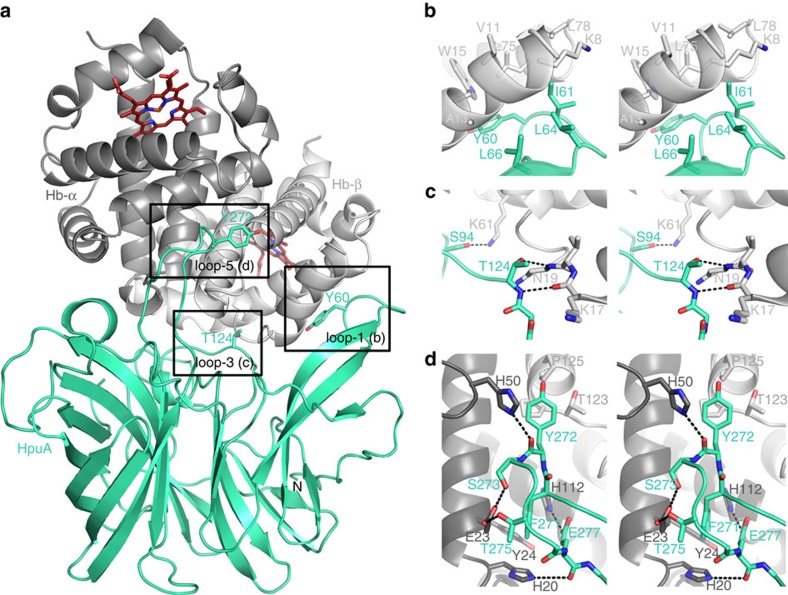
Crystal structure of the KdHpuA:Hb complex. (**a**) Cartoon representation of half of the asymmetric unit, showing a KdHpuA monomer (cyan oriented as in Fig. 1b) binding to an α/β dimer of Hb (darker and lighter grey, respectively). The two haem groups of the Hb dimer (red) and key residues on four of the KdHpuA loops are shown as stick representations. (**b**) Close-up view in stereo of the interaction between KdHpuA loop-1 and Hb. Stick representations of important residues on both sides of the interface are shown. Carbon atoms are coloured as in **a**, oxygen atoms are red and nitrogen blue. (**c**) Stereo view of the interaction between KdHpuA loop-2 and loop-3, and Hb. Representations as in **b** with hydrogen bonds are shown as black dashed lines. (**d**) Stereo view of the interaction between KdHpuA loop-5 and Hb. Representation is conserved from **a** to **c**. A full list of interacting residues can be found in Supplementary Table 1.

Crystallization of KdHpuA and Hb at a ratio of one HpuA molecule per Hb dimer yielded crystals that diffracted to 2.3 Å (Table 1 and Supplementary Fig. 1c). We solved the structure by molecular replacement using KdHpuA and oxygenated human Hb as models and observed a tetramer of Hb bound to two molecules of HpuA in the asymmetric unit. Each HpuA molecule binds to an Hb dimer and makes contact with both the α and β Hb chains (Fig. 4 and Supplementary Table 1) and the two HpuA:Hb_2_ complexes in the asymmetric unit are nearly identical. The aforementioned loops of HpuA are major sites of interaction; loop-1 contacts the β-chain of Hb with the hydrophobic side chains of KdHpuA Tyr-60 and Ile-61 packing against Hb-β Trp-15 and Leu-75 (Fig. 4b). KdHpuA Leu-64 and Leu-66 side chains are also buried within the interface with Hb (Fig. 4b). Loop-5 interacts with the αHb chain, burying further hydrophobic side chains (Phe-271 and Tyr-272) and forming several hydrogen bonds, including between His-20 and His-50 of Hb-α and backbone carbonyls of KdHpuA, between Hb-α Glu-23 and KdHpuA Ser-273, and Thr-275 and a salt bridge between Hb-α His-112 and KdHpuA Glu-277 (Fig. 4d). Additional interactions involve KdHpuA residues on two other loops with side chains of Ser-94 and Thr-124, making hydrogen bonds with Hb-β Lys-61 side chain and Asn-19 backbone amine, respectively (Fig. 4c). Overall, 2,540 Å^2^ and 2,365 Å^2^ of surface area are buried in the interfaces between the Hb tetramer and KdHpuA chains A and B, respectively. Interestingly, the hydrogen bond density at the interface is just one per 350 Å^2^, considerably less than the overall average of ∼1 per 200 Å^2^ observed from comparing multiple complexes^34^,^35^.

The N terminus of KdHpuA when bound to Hb (labelled ‘N' in Fig. 4a) is oriented such that the haem group from β-Hb is likely to be closest to the membrane and therefore available to HpuB. The HpuA-binding site on the Hb dimer resides on the opposite side of the molecule to the observed Hp-binding site^36^, suggesting that Hb:Hp would bind HpuA equally well as Hb, and that there would be no direct binding of HpuA to Hp (Supplementary Fig. 4b).

There is little conformational change observed on KdHpuA binding to Hb (Supplementary Fig. 5). The largest single movement is of loop-1, the tip of which moves 6.2 Å; however, the conformation of the loop remains unchanged and a pivot at the base of the long β3 and β4 strands facilitates this movement (Supplementary Fig. 5). There is also a significant displacement of loop-3 containing Thr-124, which adopts a different conformation, resulting in a 4.0-Å movement of the Thr-124 Cα (Supplementary Fig. 5). The Tyr-272 containing loop-5 moves a shorter distance (2.4 Å) and, similar to loop-1, retains the apo KdHpuA conformation when bound to Hb (Supplementary Fig. 5). Although loop-1 and loop-5 are involved in crystal contacts in the apo KdHpuA structure, suggesting crystal packing could cause the observed movement, the Thr-124 loop is free and the movement is a direct result of Hb binding. The B-factors of Gln-123 to Thr-126 of KdHpuA in the unliganded structure are higher than for any other amino acid; further suggesting this is a flexible part of the molecule. Interestingly, the Thr-124 region occurs immediately after an absolutely conserved Cys–Cys motif (KdHpuA residues 119 and 120) also found in the same location of the C-lobes of TbpB and LbpB. Strikingly, although the Cys–Cys motif of TbpB C-lobe has always been observed to form a disulphide bond, in our HpuA structures this motif is in reduced form despite a lack of reducing agent in the protein storage or crystallization buffers (Supplementary Fig. 6).

The structure of the HpuA-bound Hb shows no significant differences with published structures (Supplementary Figs 4 and 5)^37^,^38^,^39^. Regardless of the state of Hb, whether oxygenated or deoxygenated, tetrameric or bound to Hp, the residues bound by HpuA occupy very similar positions, suggesting HpuA is able to bind all forms of Hb as observed in pull downs using Hb or Hb:Hp beads (Fig. 3 and Supplementary Fig. 4). The quaternary structure of the Hb tetramer in our crystals is identical to the R2 state observed previously in Hb crystals grown in low-salt conditions (Supplementary Fig. 7)^37^. Solution nuclear magnetic resonance (NMR) evidence suggests oxygenated Hb exists in equilibrium between R and R2 states and, therefore, we do not believe HpuA binding has significantly altered the Hb structure^40^.

It is worth noting that, although the C-lobe of TbpB is structurally more closely related to HpuA, it is possible to superpose KdHpuA on the transferrin-bound TbpB N-lobe (Supplementary Fig. 3b,c,g). However, such a superposition does not result in an overlap of the Hb- and transferrin-binding sites, demonstrating the high degree of divergence between these proteins. Likewise, the Hb-binding site of HpuA is on a different surface of the fold to the Factor H-binding surface of Factor H-binding protein (Supplementary Fig. 3h).

### Mutating the Hb interaction site on KdHpuA reduces binding

**Figure 5:**
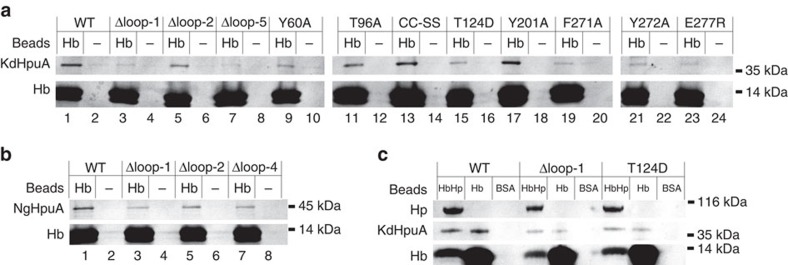
The affect of HpuA mutations on pull down by Hb. (**a**) Coomassie-stained gel showing pull-down results of Hb and control (−) beads with wild type (WT) and mutated KdHpuA protein including deletion mutants, single point mutants and mutation of the Cys–Cys motif (residues 119–120) to Ser–Ser (CC–SS). (**b**) Pull-down results with Hb and control beads pulling down NgHpuA wild type and loop deletions. (**c**) Binding of KdHpuA wild type and mutants to HbHp beads, a similar loss of binding is observed as compared with Hb beads alone. The positions of molecular weight markers are shown to the right of each gel slice. Input samples from these pull downs are shown in Supplementary Fig. 8.

To validate the HpuA:Hb interface observed in our crystal structure, we designed KdHpuA mutants targeting the Hb-binding loops and tested their binding by pull-down assay. Deleting either of the KdHpuA loops that make extensive interactions with Hb (loop-1, residues 58–64 or loop-2, residues 270–277 replaced with Gly–Ser–Gly) severely reduced the amount of HpuA pulled down by Hb (Fig. 5a, lanes 3 and 7), but did not significantly alter the folded structure of the protein as assessed by one-dimensional ^1^H NMR spectroscopy (Supplementary Fig. 9a). Mutating either of the key aromatic residues on loop-5 (Phe-271 or Tyr-272) to alanine or mutating Glu-277 to arginine also reduced the amount of HpuA pulled down, as did, to a lesser extent, mutating Tyr-60 to alanine (Fig. 5a, lanes 19, 21, 23 and 9). Introducing an aspartate side chain at the site of Thr-124 to oppose a negative surface on Hb led to a significant decrease in the HpuA pull down (Fig. 5a, lane 15). Deleting loop-2 containing Ser-94 decreased the apparent affinity of KdHpuA for Hb but had a smaller impact than deletion of either loop-1 or loop-5 (Fig. 5a, compare lane 5 with lanes 1, 3 and 7). Likewise, introducing a single Thr-96 to alanine mutation in this loop had a minimal effect on the apparent affinity (Fig. 5a, lane 11). Mutating the singly hydrophobic residue at the tip of the disordered loop-4 from residues 198 to 205 (Tyr-201 to alanine) had no effect on binding as assessed by pull down (Fig. 5a, lane 17). The Tyr-201 loop is oriented towards Hb but does not make direct contact; notably, this loop-4 is three residues longer in the *N. gonorrhoeae* homologue and contains three hydrophobic residues (Tyr-226, Tyr-227 and Leu-230), two of which are conserved in *N. meningitidis* (Fig. 2b). Both cysteine residues of the conserved Cys–Cys motif can be mutated to serine (CC–SS) without affecting the apparent affinity of HpuA for Hb (Fig. 5a, lane 13). Reduced binding to Hb:Hp beads was observed for T124D and Δloop-1 HpuA proteins with the pattern of binding, reflecting that observed with Hb beads (Fig. 5c).

**Figure 6:**
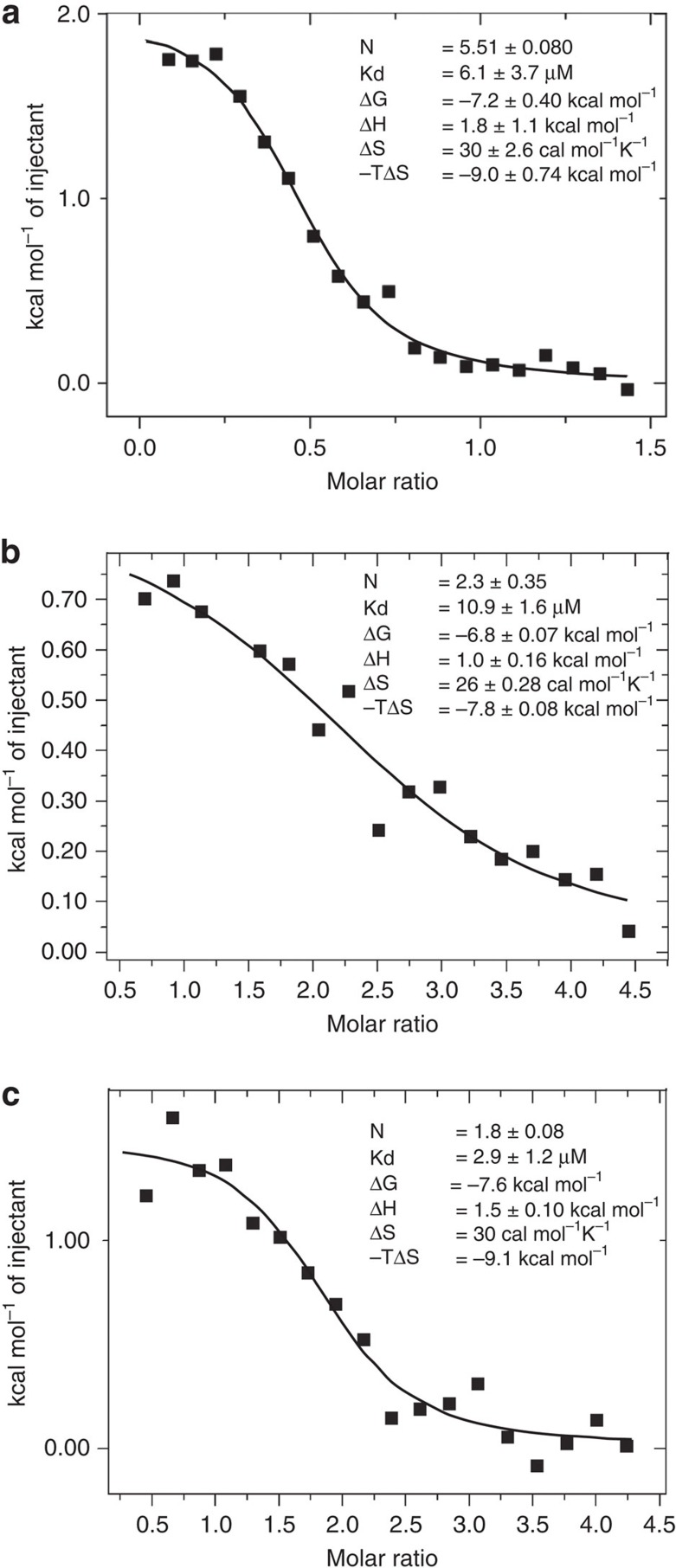
Isothermal titration calorimetry analysis of Hb binding to KdHpuA. (**a**) Multiple 2 μl injections of 1.3 mM Hb were titrated into 170 μM KdHpuA. Values given for the stoichiometry, affinity and thermodynamics of the interaction are means and s.d. of five independent experiments. (**b**) The reverse experiment to **a**—2 μl injections of wild-type KdHpuA at 600 μM were titrated into Hb at 24 μM. Binding values are means and s.d. of two independent experiments. (**c**) KdHpuA at 800 μM titrated into 39 μM Hb:Hp(1–1), values are calculated from fitting to a single experiment. In all cases a similar titration of injectant into buffer was subtracted before analysis. (See Supplementary Fig. 10 for the raw data.).

To further investigate the interaction we pursued a more quantitative assay and measured the affinity of the interaction using isothermal titration calorimetry (ITC). Titrating Hb into KdHpuA gave an apparent dissociation constant of 6.1 μM (after carefully controlling for the tetramer/dimer dissociation of Hb; Fig. 6a and Supplementary Fig. 10a). In agreement with the structure showing only seven hydrogen bonds and a single salt bridge, but large buried hydrophobic surfaces, the interaction is entropically driven and endothermic. The stoichiometry as measured by ITC (*N*=0.5) also agrees with the crystal structure with 0.5 Hb tetramers binding to each HpuA molecule. ITC measurements of KdHpuA mutants T96A, Y201A and CC–SS binding to Hb showed no change in affinity, but no heats of binding could be detected for any other mutants above the background Hb tetramer dissociation. Interestingly, we found the presence of a reducing agent during overnight dialysis before the ITC experiments to be essential for maintaining proteins competent for interacting. Presumably at these high concentrations (above 1 mM), oxygenated Hb will be subject to oxidative damage overnight at 4 °C. One site that has previously been observed to undergo oxidative damage, Hb-β Trp-15 (ref. 41), is a key residue at the interface where it interacts with KdHpuA Tyr-60 (Fig. 4b). Despite the solubility limit of HpuA preventing collection of a full sigmoidal curve, the reverse titration (HpuA into Hb) confirmed the approximate affinity, stoichiometry and thermodynamics of the interaction, showing as it did, an endothermic 2:1 HpuA:tetrameric Hb interaction with a dissociation constant of 10.9 μM (Fig. 6b and Supplementary Fig. 10b). ITC analysis of HpuA similarly titrated into Hb:Hp (using 1–1 Hp to ensure a homogenous Hb:Hp complex) again revealed a comparable interaction, with two HpuA molecules binding to each Hb_4_:Hp_4_ complex in an endothermic binding event with suggestion of a slightly tighter binding (2.9 μM apparent *K*_d_) (Fig. 6c and Supplementary Fig. 10c).

**Figure 7:**
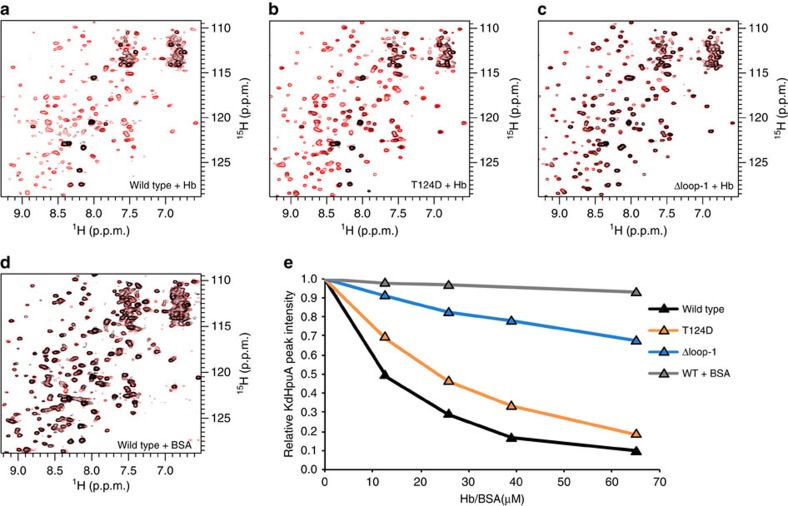
NMR spectroscopy analysis of Hb binding to KdHpuA. HSQC spectra were collected of 130 μM wild type, T124D and Δloop-1 KdHpuA alone, and with 13, 26, 39 and 65 μM Hb or BSA. (**a**–**d**) HSQC spectra of the start (0 μM Hb/BSA—red) and end (65 μM Hb/BSA—black) points of titration of Hb into wild type (**a**), Hb into T124D (**b**), Hb into Δloop-1 (**c**) and BSA into wild type (**d**). (**e**) Chart showing the relative intensities of KdHpuA amide peaks as they decrease during titration with Hb but not with BSA.

Further validation of the interface observed in the crystal structure was obtained through solution NMR spectroscopy. An ^1^H–^15^N heteronuclear single quantum coherence (HSQC) NMR spectrum was acquired on ^15^N-labelled wild-type KdHpuA at 130 μM and subsequent titration with Hb led to a stepwise decrease in peak intensities as the labelled protein was incorporated into a 132-kDa KdHpuA_2_:Hbα_2_β_2_ complex (Fig. 7a). A few of the most intense peaks of the spectrum remain undiminished throughout the titration and were not included in the following calculations. We interpret these peaks to correspond to residues of the unstructured N terminus of the protein, which tumble independently from the core of the protein and are therefore not broadened similar to the rest of the protein in response to the slower tumbling of the larger complex. On addition of 0.3 molar equivalents of Hb, KdHpuA amide peaks diminished to 16% of their original height and further dropped to 10% after addition of 0.5 molar equivalents (Fig. 7a,e). No reduction in peak intensity was observed when BSA was titrated into KdHpuA (Fig.7d,e). A similar titration using ^15^N-labelled KdHpuA T124D at the same concentration showed reduced binding to Hb: 0.5 molar equivalents of Hb were required to reduce the peaks to 19% of their original height (Fig. 7a,c). In a third experiment using KdHpuA Δloop-1, 0.5 molar equivalents of Hb could be added and the measured HpuA amide peak heights remained at 58% of the original value (Fig. 7a,d). These data are in agreement with the pull-down results showing a moderate reduction in Hb binding for KdHpuA T124D and a more severe decrease in Hb affinity of KdHpuA Δloop-1. Although an accurate dissociation constant determination is not possible from this data due to the lack of a clear end point (signal disappears below the noise level rather than shifts to a new position) and because not all HpuA is visible in the initial spectra due to aggregation, estimates for *K*_d_ values are 10 μM for wild-type KdHpuA, 16 μM for T124D and 150 μM for Δloop-1. Notably, in the absence of Hb, the HSQC spectra of the mutant proteins showed little difference to the wild type, further demonstrating the mutations caused no major alterations to the overall protein structure.

### HpuA homologues share the same Hb-binding interface

We probed the conservation of the observed HpuA:Hb interface by introducing mutations at key sites in NgHpuA and measuring relative affinity for Hb using pull-down assays. Without a high-resolution structure of the NgHpuA:Hb complex, designing single point mutations to abolish binding proved challenging. However, deleting loop-1 (residues 77–82, equivalent to KdHpuA residues 60–65), loop-2 (residues 111–116, KdHpuA equivalent 94–99) or loop-4 (residues 225–231, KdHpuA equivalent 201–204) of NgHpuA and replacing with Gly–Gly–Gly sequences led to significantly reduced binding to Hb resin (Fig. 5b, lanes 3, 5 and 7). Deletion of loop-1 had the most significant effect (Fig. 5b, lane 3), with a less pronounced loss of affinity resulting from deleting loop-2 (Fig. 5b, lane 5), which also showed a minor effect for KdHpuA, and loop-4 (Fig. 5b, lane 7), which does not form a direct interaction with Hb in the KdHpuA-Hb crystal structure. NMR spectroscopy again confirmed that the structures of the wild type and mutants are not significantly affected by the deletions (Supplementary Fig. 9b). The effect of deleting loop-4 is particularly interesting as the equivalent region of KdHpuA, albeit being three residues shorter, is not observed to interact directly with Hb and mutation of the single hydrophobic residue on this loop for KdHpuA had no effect on binding affinity.

## Discussion

In this work we have identified for the first time a direct interaction between HpuA and Hb, have described the structure of the complex in detail and have shown that the interaction is conserved in homologues across the diverse *Neisseriaceae* family. In the absence of HpuB, direct binding of HpuA to Hb on the surface of *Neisseria* was not previously detected. The disparity between this and our work may be explained by a lack of sensitivity of the cell-based assays or alternatively HpuA on the cell surface in the absence of HpuB may be incorrectly oriented and restrained such that Hb binding is precluded. The loops identified here to be important for HpuA binding to Hb are under significant immune selection in *Neisseria*^25^, consistent with their highly exposed localization attracting attention of the host defenses and thereby promoting rapid evolution through genetic exchange^42^. The lack of conservation of Hb-binding residues in HpuA homologues and yet the retention of Hb binding is intriguing and would suggest this is an important feature of the protein. Our results therefore prove that a major function of HpuA is to contribute to the binding affinity of bacterial cells to Hb via this direct interaction. Conventionally, it is expected that functional residues of a protein remain conserved; however, it would appear that for HpuA the selective pressure applied to the functional loops by the host immune system prevents this. Nevertheless, we propose that a variety of hydrophobic residues in strategically important locations are sufficient to retain Hb binding. It is conceivable that some HpuA homologues compensate for loss of Hb-binding residues in one loop by extending other loops. For example, NgHpuA has a shorter loop-5 than that of KdHpuA, but potentially forms extra interactions from a longer loop-4 containing two tyrosine side chains and one leucine (Fig. 2b). Indeed, deleting loop-4 of NgHpuA does affect Hb-binding affinity (Fig. 5b). The extraordinary ability of *Neisseriaceae* to vary functional regions of HpuA without disrupting the base function suggests the use of this protein in a vaccine would probably be challenging. Similar variation has been observed in transferrin-binding residues of TbpB (ref. 43).

The stoichiometry of one HpuA molecule per Hb dimer observed in the crystal structure and suggested in solution by ITC is biologically logical, given that the most probable source of Hb is in Hb:Hp complexes wherein Hb is dimeric. Moreover, the predicted position of the membrane relative to the HpuA:Hb structure has significant implications for the mechanism of haem uptake. In the orientation observed in the crystal structure, with loop-1 binding Hb-β and loop-5 binding Hb-α, the N terminus of KdHpuA would be oriented such that the haem group of Hb-β is proximal to the membrane and therefore the HpuB import channel (Supplementary Fig. 11). In this orientation, and with insufficient conformational freedom to allow the protein to rotate 180° relative to the membrane, it is tempting to speculate that only the haem group of Hb-β would be imported. However, owing to the high degree of similarity between Hb-α and Hb-β, we cannot discount the possibility that HpuA can bind Hb in the opposite orientation, but that crystallization has trapped just one conformation. Alternatively, the complex could be rotated 90° to the membrane presenting either haem group to HpuB. Finally, for pathogenic *Neisseria* species, HpuA homologues have significantly longer N-terminal sequences, which could potentially confer more conformational flexibility with respect to the membrane (Fig. 2b).

In comparison with the low micromolar binding observed here between HpuA and Hb, the interaction between transferrin and TbpB is significantly higher (reported *K*_d_ ranges from 7 to 60 nM (refs 27, 44)). Although the comparative weak binding of the partial receptor for Hb is understandable, given that the *K*_d_ of the whole HpuAB receptor for Hb has been reported to be 150 nM (ref. 24) (compared with a *K*_d_ of 0.8 nm for TbpAB-binding transferrin^44^ and sub- to low-nanomolar affinities of siderophore receptors for their ligands^45^,^46^), it bears discussion as to its functional significance. The variation in binding affinity may reflect the relative abundance of the iron bound ligands of the TonB-dependent receptors in question and competition between the bacterial receptors and host receptors or innate immunity proteins. In healthy human serum, extracellular Hb concentrations up to 3 μM have been observed and this probably increases during infection due to increased haemolysis^47^. Significantly, both *Kingella* species and *E. corrodens* are β-haemolytic^48^,^49^ and *E. corrodens* is commonly observed in coinfections with haemolytic Gram-positive bacteria^50^,^51^. Although the meningococcus is not observed to be haemolytic, even a small amount of haemolysis as a consequence of sepsis would significantly increase serum Hb levels. For gonococci, abundant Hb is probably available during menses. To achieve minimum free iron concentrations, serum transferrin is only 30% saturated with iron, presenting would-be scavengers with a greater challenge: to bind selectively only iron bound transferrin^52^. TbpB overcomes this obstacle by binding specifically with high affinity to transferrin with iron-occupied C-lobe. No synonymous role is required for haem extraction from Hb, a protein intimately associated with its iron porphyrin cofactor. Rather, HpuAB is able to use different forms of Hb, whether free or bound by Hp, and we demonstrate HpuA exhibits promiscuous binding to different Hb forms reminiscent of the whole receptor. Importantly, as HpuA knockout *N. meningitidis* are unable to import haem from Hb, the primary role of HpuA appears to be more closely linked with iron uptake rather than Hb binding. TbpB deletion mutants can in come cases survive on transferrin iron *in vitro*^26^, suggesting the primary role for TbpB is in holo-transferrin binding but not precluding the possibility that TbpB also has a more direct, and as yet unidentified, role in uptake.

In agreement with this reasoning, the observation that mutation of the conserved Cys–Cys motif (KdHpuA residues 119 and 120) does not affect the binding of HpuA to Hb also suggests that binding to Hb is only part of the function of HpuA. As an absolutely conserved motif, both in HpuA and in the C-lobe of TbpB, but not in other extracellular lipoproteins of similar topology or in the N-lobes of TbpB and LbpB, we conclude that this motif is indeed functional and not purely structural. For TbpB, transferrin binding is localized to the N-lobe, whereas the role of the C-lobe (containing the Cys–Cys motif) is not defined. This leads us to hypothesize that both proteins are bifunctional; it is likely to be that TbpB, through gene duplication, has evolved two domains, each specialized for a specific function, whereas HpuA conducts both functions with a single domain. We can speculate that, as HpuA deletion *N. meningitidis* showed slower dissociation from Hb^23^, the second function could concern the release of the target protein (Hb or transferrin) or extraction of iron or haem as a requirement before release. Alternatively, a potential role could be in protecting nearby proteins and the outer membrane from oxidative damage. Fittingly, although this motif invariantly forms a disulphide in all published TbpB structures, none of the KdHpuA structures published here contain a disulphide (Supplementary Fig. 6), suggesting the Cys–Cys motif in HpuA and TbpB could be redox active. In the KdHpuA apo structure, the region is close to a crystal contact, is poorly defined and probably exists in different conformations, the predominant of which does not include a disulphide; however, in the HpuA:Hb structure, the region is very well defined and neither chain A nor chain B contains a disulphide. The published crystal structures of TbpB:transferrin and TbpA:transferrin, and the electron microscopy structure of the tertiary TbpA:TbpB:transferrin complex do not suggest a function for the C-lobe, as these structures capture a single state with TbpB N-lobe bound to transferrin^28^,^29^. We propose that future structural information from a HpuA:HpuB:Hb heterotrimer would help to elucidate the second role of these proteins by visualizing the location of the Cys–Cys motif in the context of its TonB-dependent receptor and target.

In conclusion, the structures reported herein, combined with the biochemical evidence of their relevance, provide a robust foundation for a deeper understanding of haem uptake from Hb by *Neisseriaceae* and add to our knowledge of host–pathogen interactions underlying several important human infections.

## Methods

### Protein expression and purification

Gonococcal HpuA from codon 6 to 343 was amplified from *N. gonorrhoeae* FA1090 genomic DNA and ligated into the pCPH6P vector^53^ between XmaI and BamHI sites, to produce pHiSH-NgHpuA. Similarly, amplification of the gene from codon 188 to 343 created pHiSH-NgHpuA-C. A synthetic gene encoding *K. denitrificans* HpuA, codon optimized for *Escherichia coli* expression, was procured from GeneArt (Life Sciences) and a construct containing codons 6–322 was ligated into the same vector to produce pHiSH-KdHpuA. Mutants in pHiSH-NgHpuA and pHiSH-KdHpuA were constructed using the QuikChange procedure (Stratagene).

Protein expression was induced in PC2 cells^53^ by addition of 0.5 mM isopropyl-β-D-thiogalactoside at A_600_ of 0.8 for 16 h at 18 °C. Harvested cells were resuspended in 10 ml per 1-litre culture volume of Buffer A (50 mM Tris/HCl pH 7.5, 500 mM NaCl, 20 mM imidazole) and stored at −80 °C. Thawed cell suspensions were supplemented with 0.5 mM phenylmethylsulfonyl fluoride and lysed by sonication. Lysates were clarified by centrifugation and incubated with mixing for 1 h at 4 °C with 3 ml NiNTA resin equilibrated in Buffer A. Following extensive washing with Buffer A, protein was eluted from the resin with 10 ml Buffer B (Buffer A with 200 mM imidazole). Prescision protease was added to the eluate at a ratio of 1 mg protease to 30 mg HpuA together with 10 mM dithiothreitol and incubated overnight at 4 °C. After concentration to 5 ml, the protein was further purified on an S75 Superdex column in Buffer C (50 mM Tris/HCl pH 7.5, 150 mM NaCl). HpuA containing fractions were pooled and concentrated to ∼10 mg ml^−1^, supplemented with 10% (v/v) glycerol, flash frozen in liquid nitrogen and stored at −80 °C.

Hb was directly purified from human blood according to Perutz^54^. Briefly, red blood cells were pelleted by centrifugation and lysed by osmotic shock by washing the cells once in 1% NaCl followed by 15 min incubation in 0.5% NaCl. The NaCl concentration was adjusted to a final concentration of 2%. The cell lysates were spun down at 35 000*g* for 1 h at 4 °C before the supernatant was carefully recovered and spun down again for a further 30 min. Purified Hb was buffer exchanged using a HiPrep 26/10 desalting column (GE Healthcare) pre-equilibrated with Buffer C. Aliquots were supplemented with 10% (v/v) glycerol, flash frozen in liquid nitrogen and stored at −80 °C.

The Hb:Hp complex was prepared by mixing purified Hb with Hp 1–1 (Sigma) at a molar ratio of 3:1 and purified from excess Hb on a Superdex 200 10/300 GL column (GE Healthcare) in phosphate buffered serum (PBS).

### Crystallization and structure determination

All crystals were grown in hanging drops by vapour diffusion. KdHpuA at 10 mg ml^−1^ in Buffer C crystallized against a reservoir containing 100 mM imidazole pH 7.8, 150 mM lithium sulfate and 6% PEG 3,000 at 4 °C. Before freezing, crystals were transferred to a cryoprotectant solution containing 100 mM imidazole pH 7.8, 150 mM lithium sulfate, 6% PEG 3,000 and 20% glycerol. *N. gonorrhoeae* HpuA-C crystals were grown at 18 °C from protein at 10 mg ml^−1^ in Buffer C over reservoirs containing 100 mM HEPES pH 6.85, 1.6 M ammonium sulfate and 0.8% PEG 400 with 200 mM sodium thiocyanate as an additive. KdHpuA:Hb complexes were crystallized by mixing equal volumes of the proteins at 10 mg ml^−1^ in Buffer C and incubating on ice for 30 min. Crystals were grown against a well of solution of 100 mM HEPES pH 7.82, 18% (w/v) PEG 4,000 and 10% (v/v) isopropanol at 4 °C. The crystals were soaked in the well solution supplemented with 20% glycerol before freezing.

Data from all crystals were collected at Diamond light source (Oxfordshire, UK), KdHpuA and NgHpuA-C data were collected on beamline I04 and KdHpuA:Hb data on I04–1. Data were collected at 100 K, indexed with Mosflm and merged using Scala^55^,^56^,^57^. KdHpuA crystals were phased from a highly redundant data set at the cobalt peak energy (1.605 Å) collected from co-crystals grown in the presence of 200 mM cobalt chloride. The structure was solved using SHARP with two cobalt sites located using SHELXD^58^,^59^,^60^. Buccaneer and ARP/wARP were used to automatically build a starting model containing 277 residues^61^,^62^. The C-terminal part of KdHpuA was used as a molecular replacement model for solving the NgHpuA-C structures using Molrep^55^,^63^. The HpuA:Hb complex structure was solved by molecular replacement by Phaser using KdHpuA and oxyHb (pdb 1HHO) as search models^55^,^64^. All structures were refined using Coot, Refmac and Phenix, to produce final models with good geometry (over 95% in Ramachandran favoured regions and <0.5% outliers) and *R*_free_ values from 19.6 to 24.4% (Table 1)^55^,^65^,^66^,^67^.

### Pull downs

Protein-linked agarose was prepared by incubating 100 μg protein (Hb, BSA or Hb:Hp) with 20 μl of NHS-activated resin (Thermo Fisher) in 500 μl PBS overnight at 4 °C with gentle agitation. The resin was recovered by centrifugation and excess protein removed by extensive washing with PBS. Unreacted sites on the beads were blocked by incubating in 1 M Tris pH 7.5 for 30 min at room temperature before further PBS washes removed excess Tris. Control beads were prepared by similarly blocking with 1 M Tris pH 7.5 without prior exposure to protein. Fifty micrograms of HpuA was added to the resin in 500 μl PBS and incubated for 2 h at 4 °C with agitation. Unbound protein was removed by washing three times with 500 μl PBS before the recovered resin was resuspended in 20 μl Laemmli buffer, boiled at 95 °C for 3 min and proteins analysed by electrophoresis on a denaturing 12% polyacrylamide gel.

### Isothermal titration calorimetry

Aliquots of KdHpuA at 130–200 μM and Hb at 1.3 mM (concentration of tetramer) were dialysed overnight at 4 °C against excess PBS supplemented with 1 mM β-mercaptoethanol. Isothermal titration calorimetry was carried out at 25 °C on an iTC200 system (MicroCal). Measurements of Hb titrated into PBS with 1 mM β-mercaptoethanol were subtracted from the experiments to control for the thermodynamics of Hb tetramer to dimer dissociation. Alternatively, 600–800 μM HpuA was titrated into 20–40 μM Hb or Hb:Hp after all proteins were purified by size exclusion chromatography in PBS on a Superdex 200 10/300 GL column (GE Healthcare). A titration of HpuA into PBS was carried out as a control. Data were fitted and analysed using Origin 7 (MicroCal).

### Nuclear magnetic resonance

Proton NMR spectroscopy was performed on 50–150 μM HpuA at 25 °C in PBS, 90% H_2_O and 10% D_2_O on a Bruker AVANCE III 600-MHz instrument equipped with a cryo probe. Titration experiments were conducted in PBS, 1 mM β-mercaptoethanol, 90% H_2_O and 10% D_2_O. Sequential 5 μl titrations of 1.3 mM unlabelled Hb or BSA were added to 500 μl of 130 μM ^15^N-labelled KdHpuA up to a maximum of 25 μl or 0.5 molar equivalents. ^1^H–^15^N correlation spectra (HSQC) were collected on a Bruker AVANCE II 800-MHz instrument. For quantitative analysis, the highest 25 peaks in the wild-type KdHpuA HSQC spectrum were selected (ignoring the first seven peaks that show a fast relaxation indicative of being freely rotating separate from the protein core) and their relative heights measured in all spectra.

## Additional information

**Accession codes:** Coordinates and associated structure factors have been deposited in the protein data bank with PDB ids 5EC6 (KdHpuA), 5EE2 (NgHpuAC) and 5EE4 (KdHpuA:Hb).

**How to cite this article:** Wong, C. T. *et al*. Structural analysis of haemoglobin binding by HpuA from the *Neisseriaceae* family. *Nat. Commun.* 6:10172 doi: 10.1038/ncomms10172 (2015).

## Supplementary Material

Supplementary Information Supplementary Figures 1-11, Supplementary Table 1 and Supplementary References.

## Figures and Tables

**Table 1:** Data collection and refinement statistics.

	**KdHpuA**	**KdHpuA (cobalt SAD**)	**NgHpuA-C**	**KdHpuA:Hb complex**
*Data collection*				
Space group	P4_3_2_1_2	P4_3_2_1_2	H3	P2_1_
Cell dimensions				
*a*, *b*, *c* (Å)	102.5, 102.5, 77.2	103.4, 103.4, 77.0	61.9, 61.9, 92.2	54.8, 87.2, 124.3
*α*, *β*, *γ* (°)	90.0, 90.0, 90.0	90, 90, 90	90, 90, 120	90, 98.1, 90
Resolution (Å)	26.67–1.60 (1.69–1.60)*	28.68–1.94 (2.05–1.94)	34.97–1.95 (2.06–1.95)	41.53–2.30 (2.42–2.30)
*R*_merge_	0.102 (0.853)	0.140 (0.671)	0.084 (0.54)	0.175 (0.818)
*I*/σ*I*	12.6 (2.8)	14.9 (4.0)	9.1 (2.3)	8.6 (2.1)
Completeness (%)	100 (100)	100 (100)	99.8 (99.9)	96.9 (97.8)
Redundancy	11.4 (11.4)	18.8 (17.9)	4.0 (3.9)	6.5 (6.3)
				
*Refinement*				
Resolution (Å)	72.46–1.6		46.4–1.95	123.1–2.3
No. reflections	51939		9078	47357
*R*_work_/*R*_free_	0.171 / 0.196		0.178 / 0.23	0.202 / 0.244
No. atoms				
Protein	2278		916	8742
Ligand/ion	12		0	192
Water	328		58	374
*B*-factors				
Protein	24.79		39.00	33.55
Ligand/ion	48.53			30.05
Water	34.14		45.13	33.21
Root mean squared deviations				
Bond lengths (Å)	0.014		0.011	0.013
Bond angles (°)	1.608		1.384	1.504

SAD, single wavelength anomalous dispersion

^*^Values in parentheses are for highest-resolution shell.
