# Supplementary material for: Structural analysis of haemoglobin binding by HpuA from the Neisseriaceae family
Source: Nat Commun. 2015 Dec 16;6:10172. doi: 10.1038/ncomms10172 (PMC4703857; doi:10.1038/ncomms10172)
Supplement: Supplementary Information — Supplementary Figures 1-11, Supplementary Table 1 and Supplementary References. [file ncomms10172-s1.pdf]

Supplementary Figures

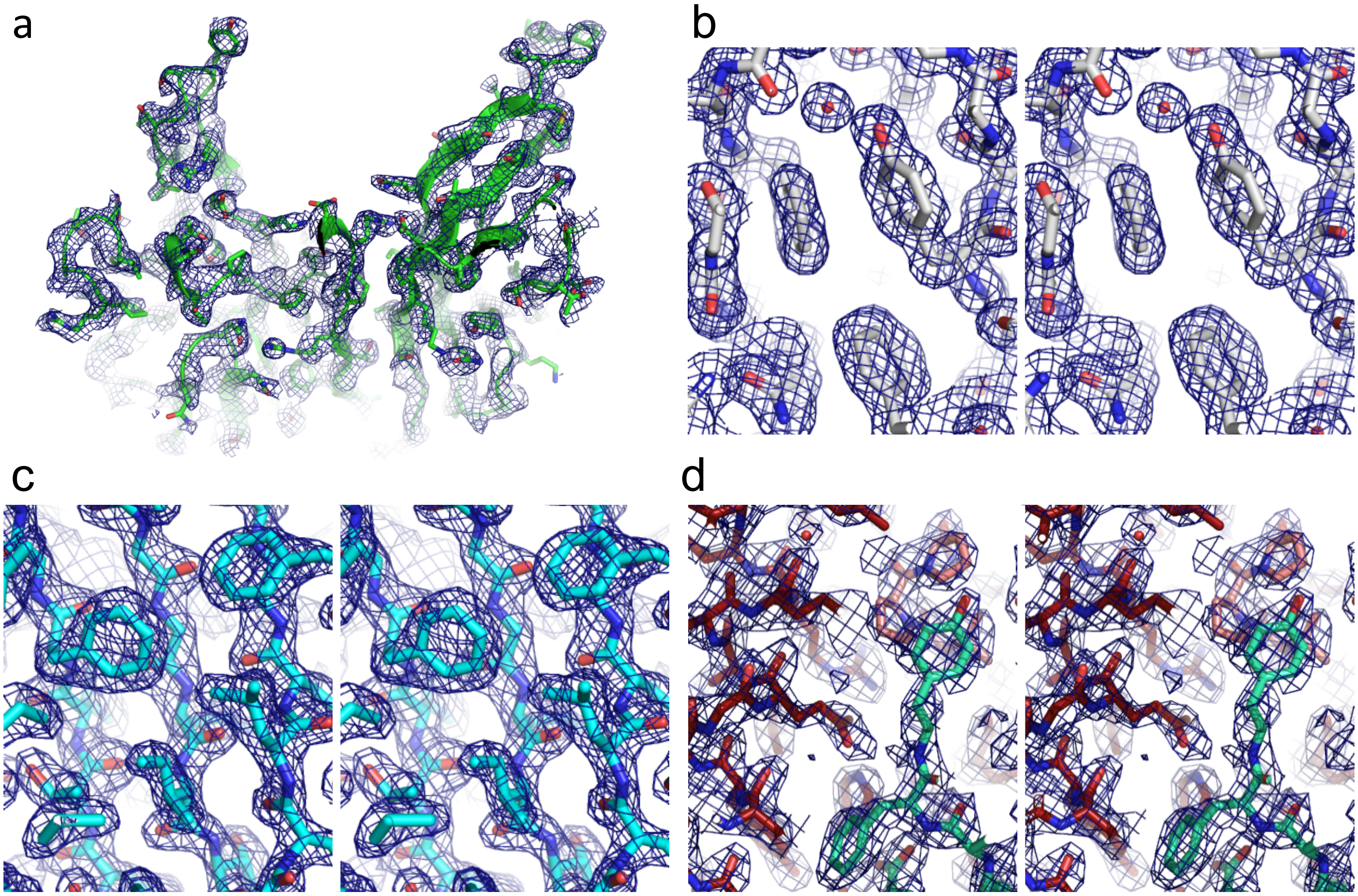

Supplementary Figure 1 Final atomic coordinates fitted into electron density maps. (**a**) Cartoon and stick representation of the KdHpuA structure in experimental $2F_O$-F_C electron density after phasing and solvent flattening by Sharp[1] contoured at 1 σ. (**b**) Stick representation of the final KdHpuA structure in final $2F_O$-F_C electron density map contoured at 1 σ. (**c**) Stereo view of the final structure of NgHpuA-C (cyan), fitted in the final $2F_O$-F_C electron density map contoured at 1 σ. (**d**) Stereo view of the final structure of KdHpuA (teal) and Hb (red) in complex, fitted in the final $2F_O$-F_C electron density map contoured at 1 σ.

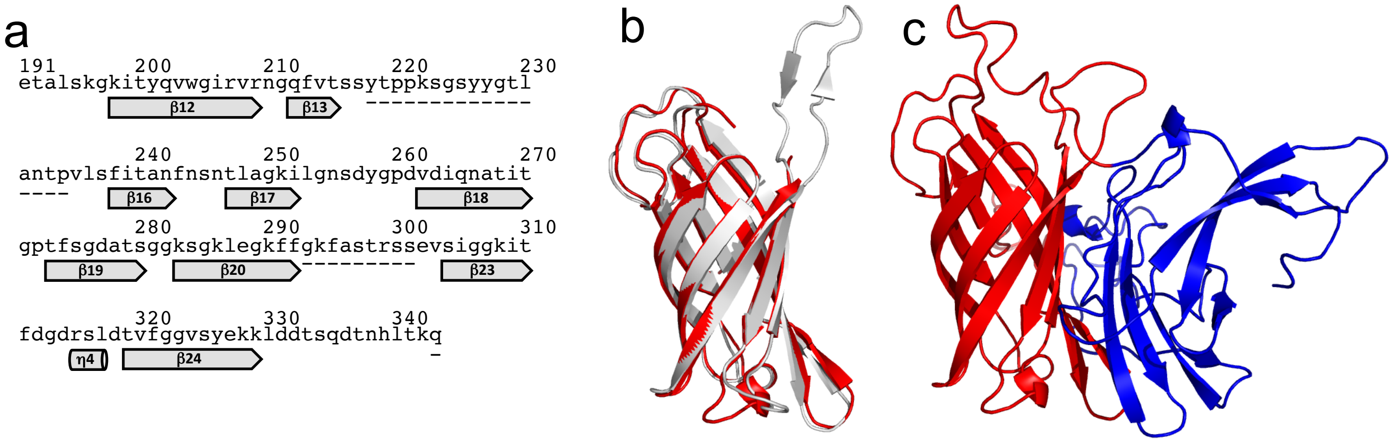

Supplementary Figure 2 The structure of *N. gonorrhoeae* HpuA. (**a**) Sequence of the construct of NgHpuA-C indicating regions of secondary structure (cylinders and arrows) and regions not visible in the electron density (dashes). (**b**) Crystal structure of NgHpuA-C (red) superposed with the equivalent region of KdHpuA (white). (**c**) Full length model of NgHpuA composed from the crystal structure of NgHpuA-C (red) with a homology model of the N-terminal sequence (blue) based on the KdHpuA structure.

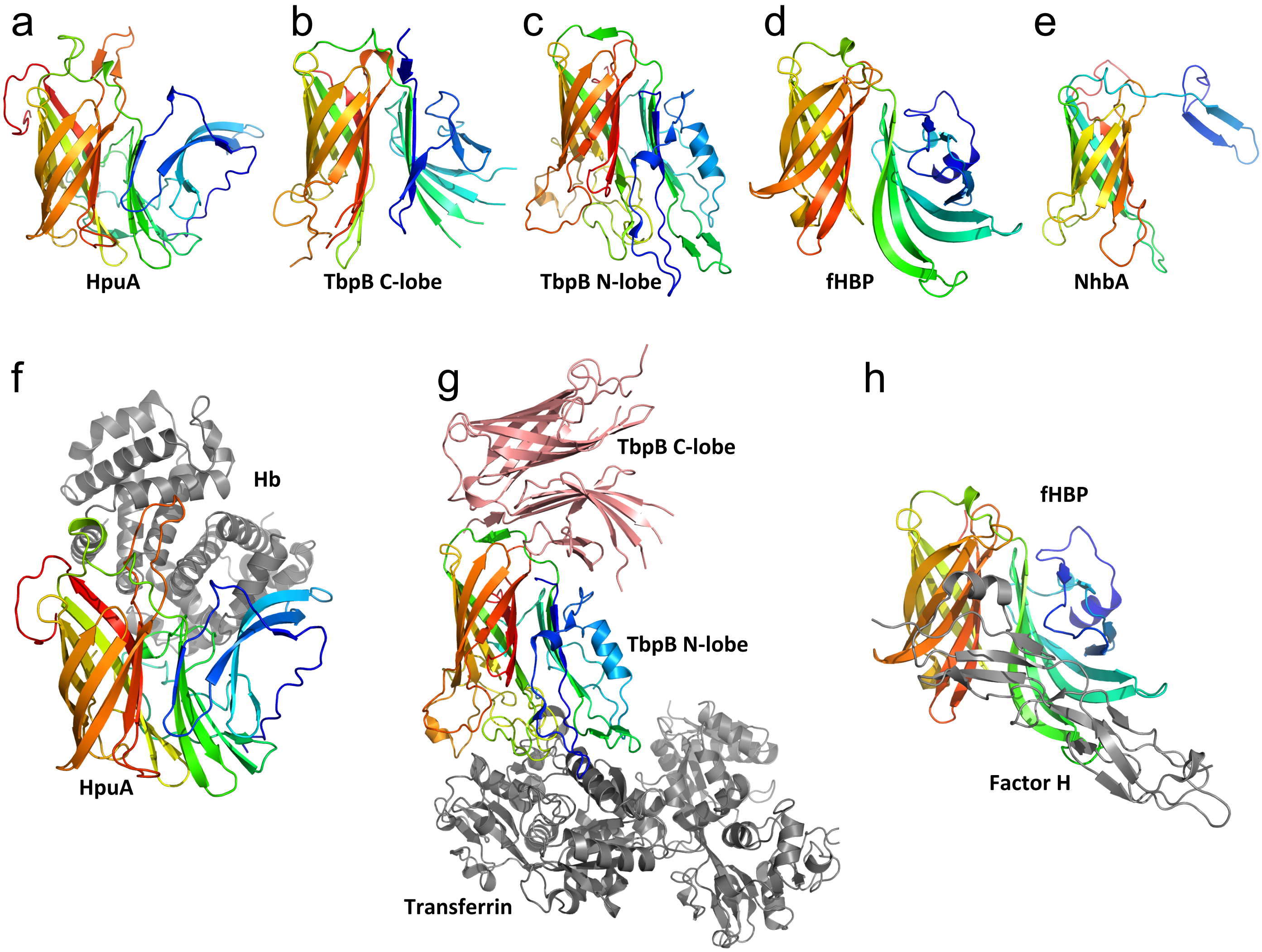

Supplementary Figure 3 Comparison of KdHpuA with TbpB (pdb id 3VE1[2]), Factor H binding protein (2W80[3]) and NhbA (2LFU[4]). Rainbow cartoon representations of HpuA (**a**), TbpB C-lobe (**b**), TbpB N-lobe (**c**), Factor H binding protein (**d**) and NhbA (**e**) structures aligned according to secondary structure elements demonstrate the conserved fold. HpuA bound to Hb (**f**), TbpB N-lobe bound to transferrin (**g**) and Factor H binding protein bound (fHBP) to Factor H (**h**) are colored and aligned as in the earlier panels to demonstrate the different binding interfaces for their respective ligands (in gray).

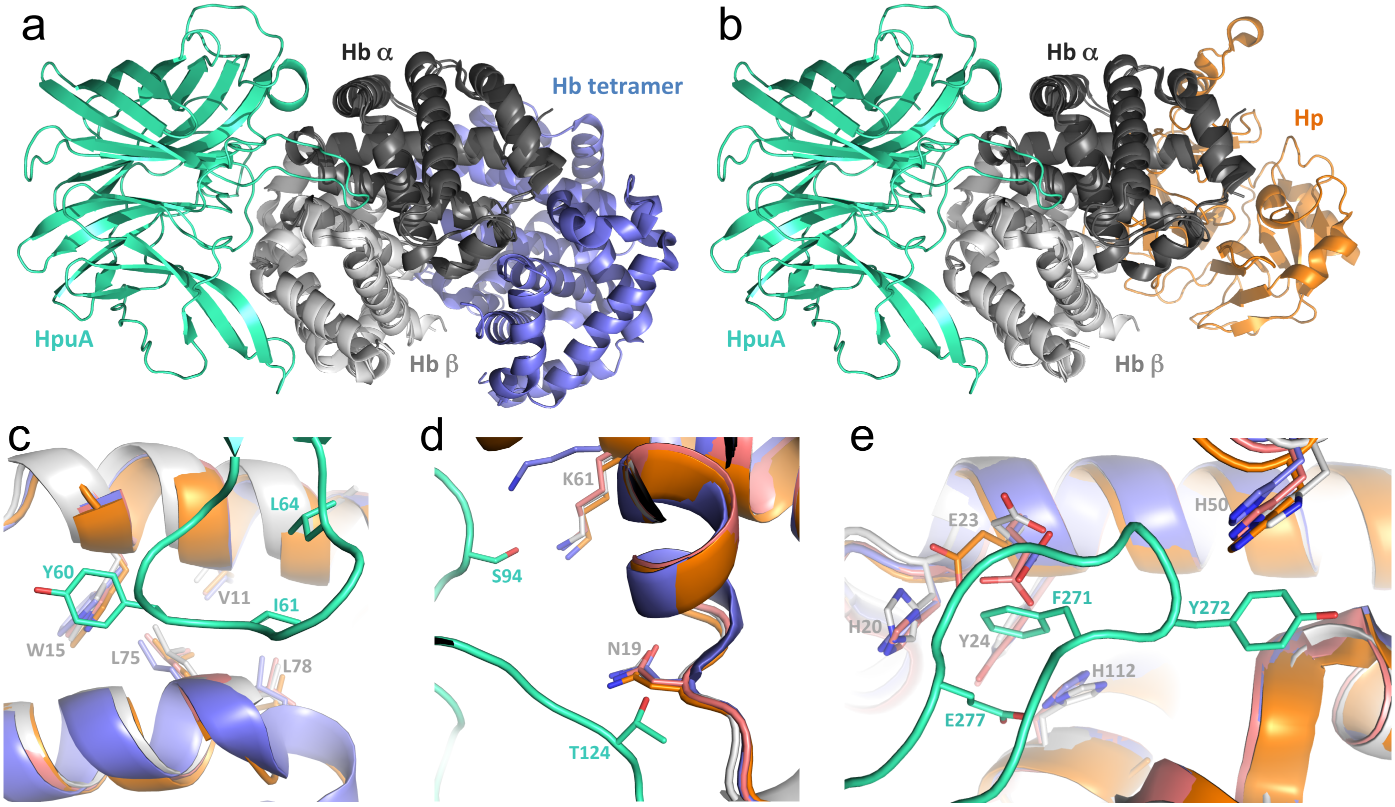

Supplementary Figure 4 HpuA binding modeled on different forms of Hb. (**a**) The HpuA:Hb structure (cyan and gray) is superposed with an isolated deoxygenated Hb tetramer structure (2dn2[5]) revealing no clash between HpuA and the second Hb dimer. (**b**) A similar alignment of HpuA:Hb onto Hb:Hp (4X0L[6]), again revealing no clash between HpuA and the host Hb binding protein. (**c**) Zoomed in view of HpuA loop-1 interacting with Hb (gray). The superposed structures of oxygenated Hb (pink, 2dn1[5]), deoxygenated Hb (blue) and Hp-bound Hb (orange) reveal no significant movement of the HpuA-interacting residues. (**d**) and (**e**) respectively show similar detailed views of the loop-3 and loop-5 interacting regions.

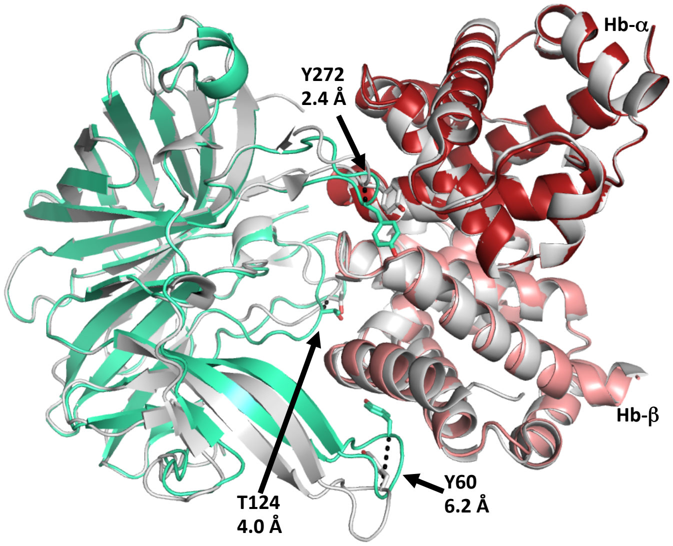

Supplementary Figure 5 Comparison of KdHpuA and Hb (pdb 1bbb[7]) structures alone (gray) and in the KdHpuA:Hb complex (cyan and red respectively). The movements of loop-1, -3 and -5 of KdHpuA are indicated by black dashes and the side chains of key residues are shown as stick representations.

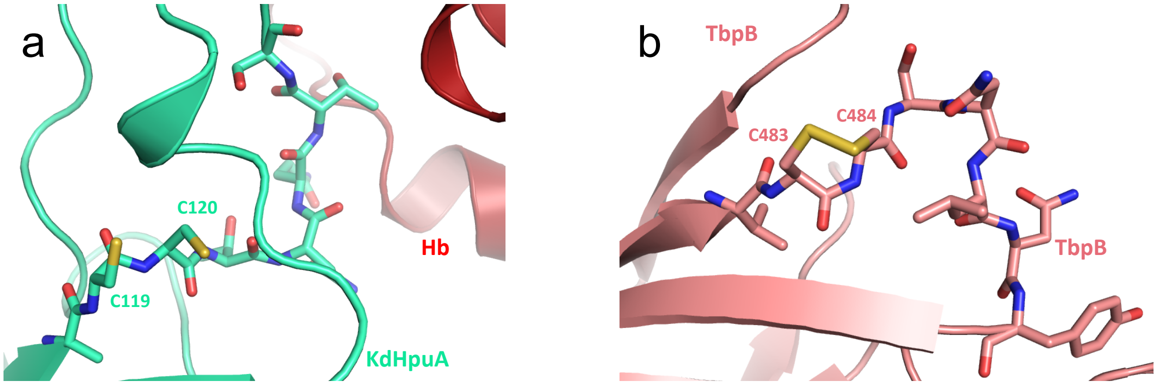

Supplementary Figure 6 Comparison of the loop containing the Cys-Cys motif of HpuA and TbpB. (**a**) Close up of loop-2 of KdHpuA (cyan), in complex with Hb (dark red). (**b**) The equivalent region of TbpB C-lobe showing the disulfide formed in the Cys-Cys motif (pdb id 3VE2[2]).

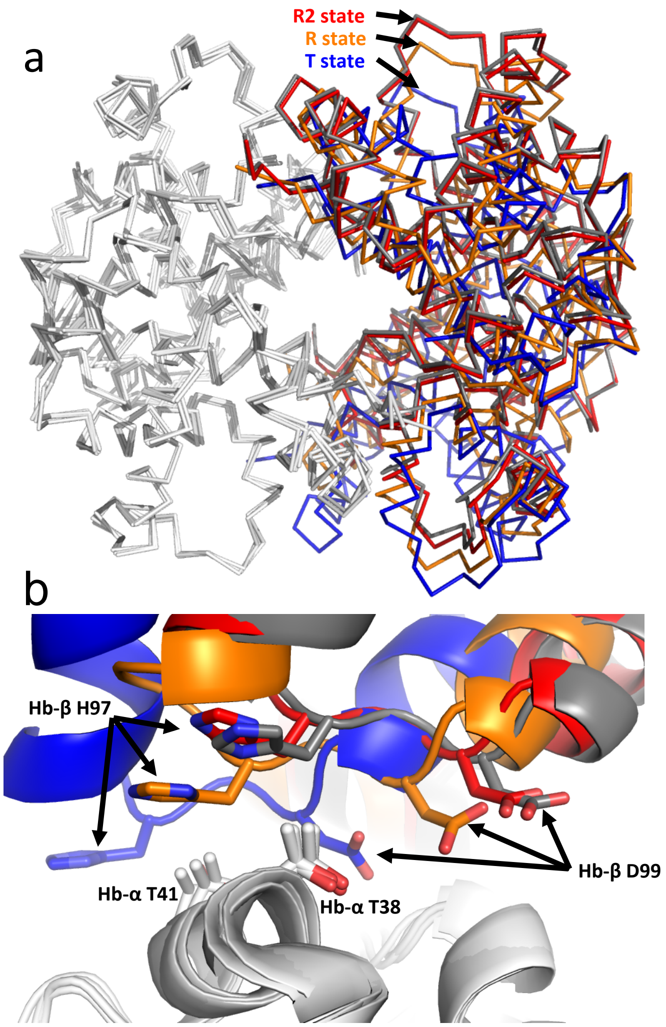

Supplementary Figure 7 The quaternary structure of Hb in complex with HpuA is the R2 state. Superposition of HpuA:Hb structure (white and gray) on Hb in the T-state (white and blue, pdb id 2dn2[5]), R state (white and orange, pdb id 2dn1[5]) and R2 state (white and red, pdb id 1bbb[7]). In both panels, one superposed α/β Hb dimer of each structure is shown in white. (**a**) Overall tetrameric structures shown as ribbon representation, the 2nd half of the Hb tetramer in complex with HpuA (gray) aligns well with the R2 state tetramer (red). (**b**) Zoomed view of the 'switch' region of the same superposed structures. Stick representations are shown of side chains important for the 'switch'.

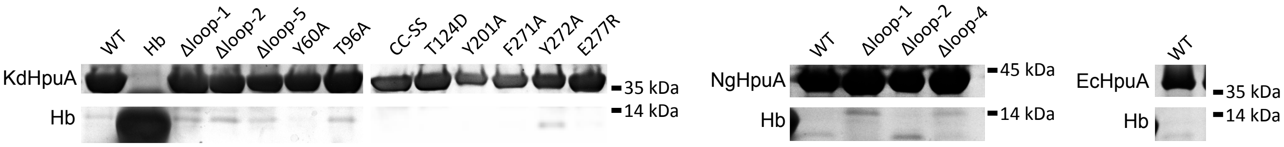

Supplementary Figure 8 Input samples from pull down experiments shown in **Figure 5**. The position of molecular weight markers are shown to the right of each gel slice.

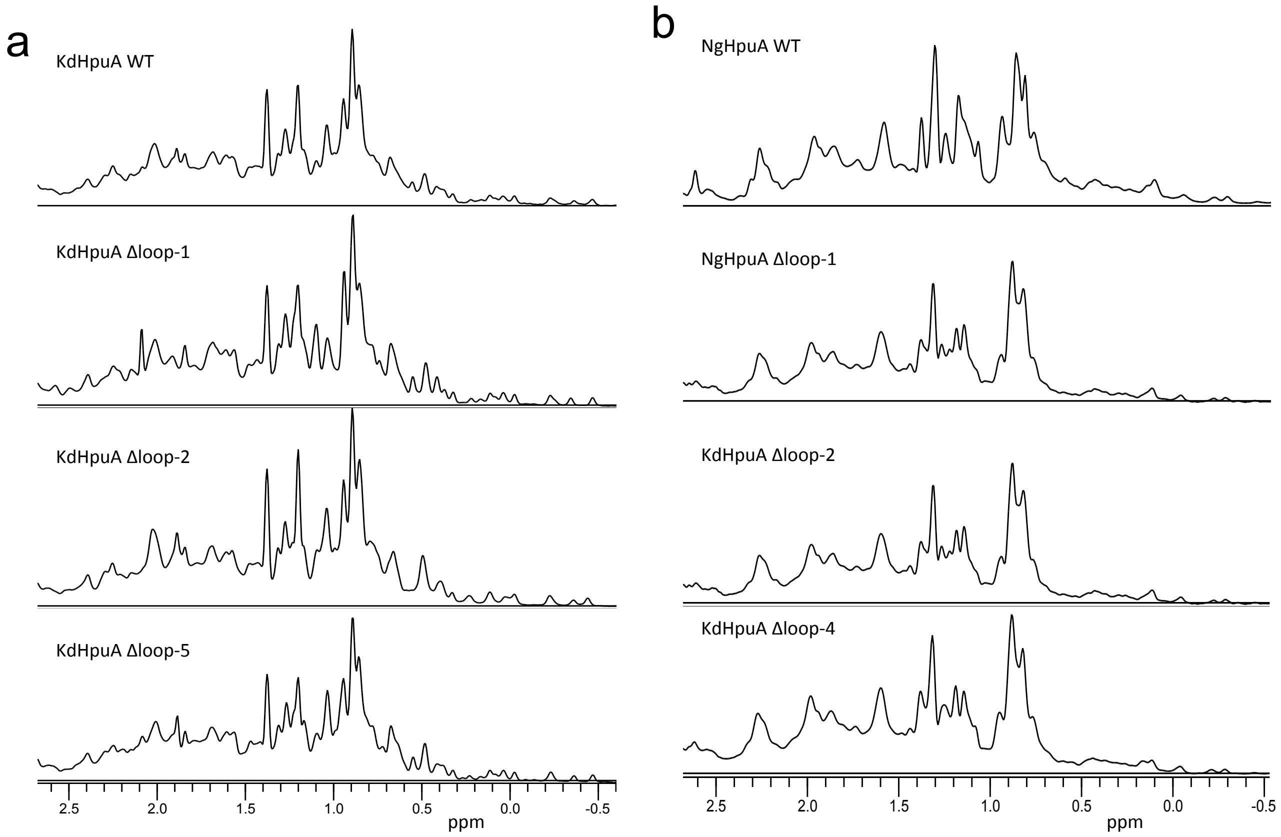

Supplementary Figure 9 Proton NMR spectra of KdHpuA and NgHpuA proteins. (**a**) Methyl region of proton NMR spectra of wild type, Δloop-1, Δloop-2 and Δloop-5 KdHpuA proteins. (**b**) Methyl region of proton NMR spectra of wild type, Δloop-1, Δloop-2 and Δloop-4 NgHpuA proteins.

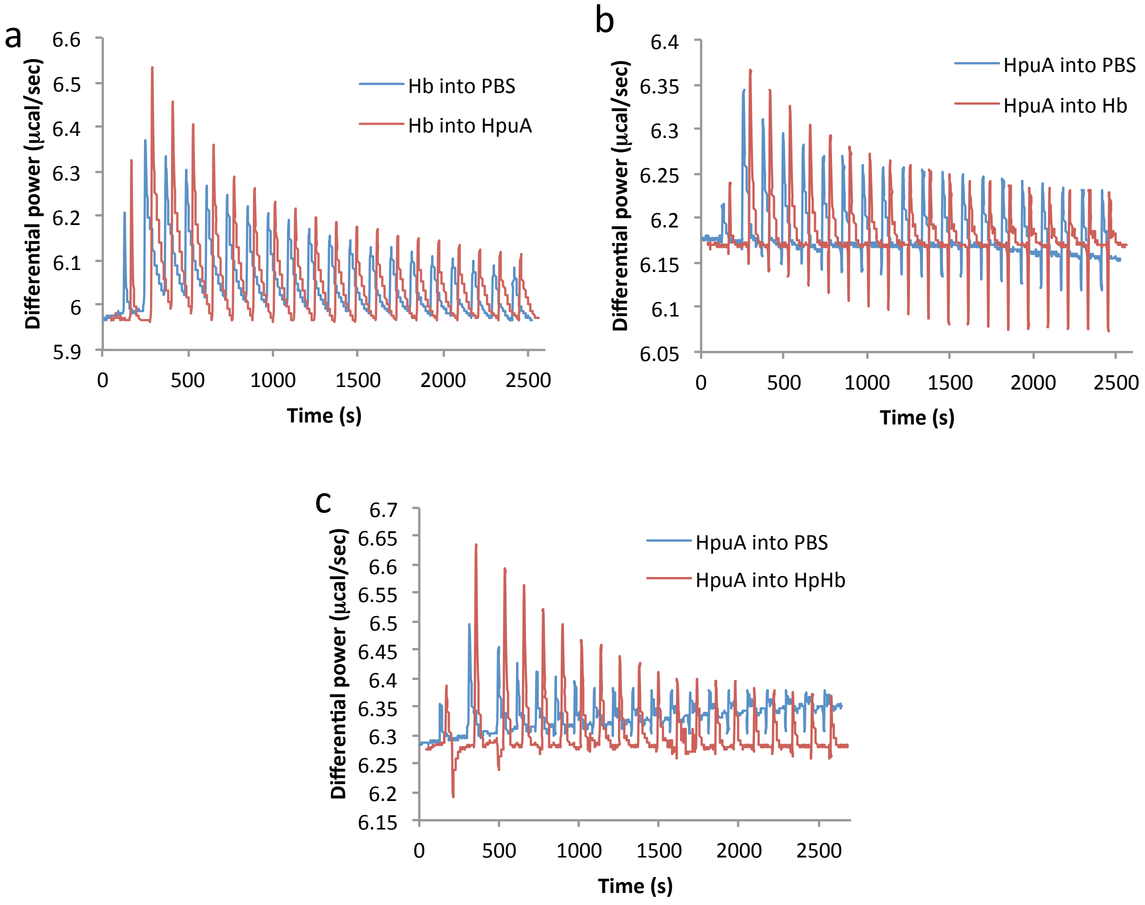

Supplementary Figure 10 Raw data from ITC experiments. (**a**) Hb titrated into KdHpuA, (**b**) KdHpuA titrated into Hb and (**c**) KdHpuA titrated into Hb:Hp. Each chart shows the experimental injections in blue and the control injections in red.

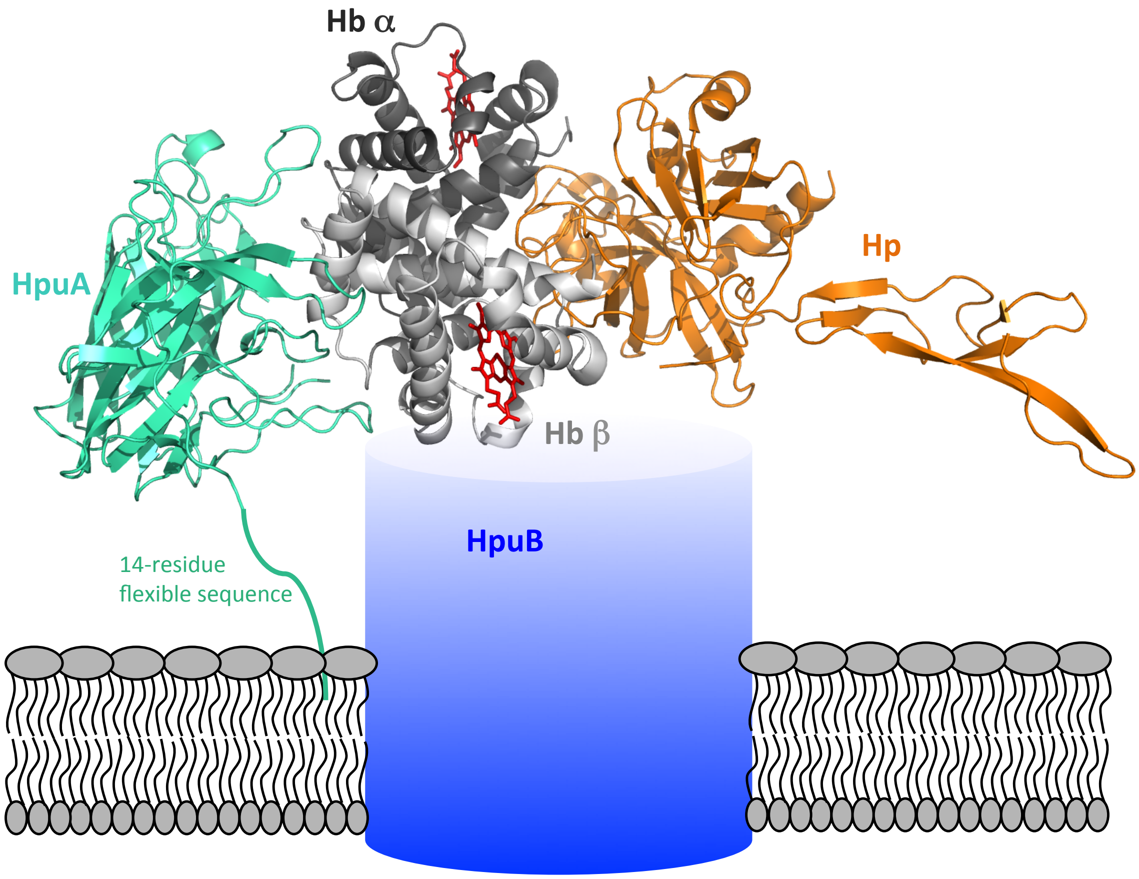

Supplementary Figure 11 Model of KdHpuA (cyan) associated with the membrane and bound to Hb (gray) and Hp (orange). The proposed position of HpuB ready to import the heme from Hb-β is shown as a blue cylinder.

Supplementary Table 1 Interactions between KdHpuA and Hb

HpuA		Hb	Distance	Type*
Loop-1	Ser58	β Thr12	2.8	vdw
	Tyr60	β Trp15	3.6	vdw
		β Ser72	3.8	vdw
		β Val20	3.3	vdw
		β Ala76	3.5	vdw
	Ile61	β Leu75	3.4	vdw
		β Thr12	3.7	vdw
	Leu64	β Thr12	3.8	vdw
	Leu66	β Thr12	3.8	vdw
		β Ser9	3.7	vdw
Loop-2	Ser94	β Lys61	2.5	H-bond
	Ser95	β Gly56	3.4	vdw
Loop-3	Asn122	β Lys17	3.8	vdw
	Gln123	β Lys17	3.4	vdw
		β Gly16	3.4	vdw
		β Val18	3.6	vdw
	Thr124	β Lys17	3.1	H-bond
		β His117	3.7	vdw
		β Val23	3.6	vdw
		β Asn19	3.2	H-bond
		β Phe118	3.6	vdw
	Ser125	β His117	3.4	vdw
	Thr126	β Glu26	3.8	vdw
		β His117	3.6	vdw
Loop-5	Phe271	α Glu23	3.6	vdw
		α Glu27	4.0	vdw
		α His112	3.3	vdw
		α Tyr24	3.7	vdw
		α His20	3.6	vdw
	Tyr272	β Pro124	3.4	vdw
		β Thr123	3.9	vdw
		β Pro125	3.4	vdw
		α Glu30	3.3	vdw
		α His50	3.7	H-bond
	Ser273	α Glu23	2.9	H-bond
	Thr275	α Glu23	2.5	H-bond
	Glu277	α His112	2.7	Salt bridge
		α His20	3.8	H-bond

* vdw = van der Waal's interaction, H-bond = hydrogen bond

Supplementary References

1. Bricogne G, Vonrhein C, Flensburg C, Schiltz M, Paciorek W. Generation, representation and flow of phase information in structure determination: recent developments in and around SHARP 2.0. *Acta Crystallogr D Biol Crystallogr* **59**, 2023-2030 (2003).

2. Calmettes C, Alcantara J, Yu RH, Schryvers AB, Moraes TF. The structural basis of transferrin sequestration by transferrin-binding protein B. *Nat Struct Mol Biol* **19**, 358-360 (2012).

3. Schneider MC*, et al.* Neisseria meningitidis recruits factor H using protein mimicry of host carbohydrates. *Nature* **458**, 890-893 (2009).

4. Esposito V*, et al.* Structure of the C-terminal domain of Neisseria heparin binding antigen (NHBA), one of the main antigens of a novel vaccine against Neisseria meningitidis. *J Biol Chem* **286**, 41767-41775 (2011).

5. Park SY, Yokoyama T, Shibayama N, Shiro Y, Tame JR. 1.25 A resolution crystal structures of human haemoglobin in the oxy, deoxy and carbonmonoxy forms. *J Mol Biol* **360**, 690-701 (2006).

6. Lane-Serff H, MacGregor P, Lowe ED, Carrington M, Higgins MK. Structural basis for ligand and innate immunity factor uptake by the trypanosome haptoglobin-haemoglobin receptor. *Elife* **3**, e05553 (2014).

7. Silva MM, Rogers PH, Arnone A. A third quaternary structure of human hemoglobin A at 1.7-A resolution. *J Biol Chem* **267**, 17248-17256 (1992).
